# Iterative Thoracentesis as First-Line Treatment of Complicated Parapneumonic Effusion

**DOI:** 10.1371/journal.pone.0084788

**Published:** 2014-01-06

**Authors:** Julien Letheulle, Pierre Tattevin, Lauren Saunders, Mallorie Kerjouan, Hervé Léna, Benoit Desrues, Yves Le Tulzo, Stéphane Jouneau

**Affiliations:** 1 Respiratory medicine department, Pontchaillou Hospital, Rennes 1 University, Rennes, France; 2 Infectious diseases and intensive care unit, Pontchaillou Hospital, Rennes 1 University, Rennes, France; 3 INSERM U835, Rennes 1 University, Rennes, France; 4 Department of medical information, Pontchaillou Hospital, Rennes 1 University, Rennes, France; 5 CIC –INSERM 0203Rennes 1 University, Rennes, France; 6 IRSET U1085, Rennes 1 University, Rennes, France; Clinica Universidad de Navarra, Spain

## Abstract

**Rationale:**

Optimal management of complicated parapneumonic effusions (CPPE) remains controversial.

**Objectives:**

to assess safety and efficacy of iterative therapeutic thoracentesis (ITTC), the first-line treatment of CPPE in Rennes University Hospital.

**Methods:**

Patients with CPPE were identified through our computerized database. We retrospectively studied all cases of CPPE initially managed with ITTC in our institution between 2001 and 2010. ITTC failure was defined by the need for additional treatment (i.e. surgery or percutaneous drainage), or death.

**Results:**

Seventy-nine consecutive patients were included. The success rate was 81% (n = 64). Only 3 patients (4%) were referred to thoracic surgery. The one-year survival rate was 88%. On multivariate analysis, microorganisms observed in pleural fluid after Gram staining and first thoracentesis volume ≥450 mL were associated with ITTC failure with adjusted odds-ratios of 7.65 [95% CI, 1.44–40.67] and 6.97 [95% CI, 1.86–26.07], respectively. The main complications of ITTC were iatrogenic pneumothorax (n = 5, 6%) and vasovagal reactions (n = 3, 4%). None of the pneumothoraces required chest tube drainage, and no hemothorax or re-expansion pulmonary edema was observed.

**Conclusions:**

Although not indicated in international recommendations, ITTC is safe and effective as first-line treatment of CPPE, with limited invasiveness.

## Introduction

Pleural infection is a common clinical problem associated with significant morbidity and mortality [1], [2]. Most guidelines recommend that complicated parapneumonic effusions (CPPE) be evacuated, in addition to appropriate antibiotics [3], but the optimal evacuation method remains controversial and poorly standardized. Current options include iterative therapeutic thoracentesis (ITTC), chest tube drainage, video-assisted thoracoscopic surgery (VATS), or thoracotomy. [4]–[6]. Few randomized studies compared evacuation methods in CPPE, but no consensus could be reached from these studies, due to their limited sample size, and heterogeneity [5], [7].

In our department, ITTC has long been the first-line treatment to remove infected pleural fluid in CPPE, in association with systemic antibiotics. The theoretical benefits associated with this procedure include shorter immobilization, and limited use of thromboprophylaxis and analgesics, as compared to chest tube drainage or surgery.

We report our experience of systematic use of ITTC as first-line treatment in CPPE, focusing on efficacy, tolerability, and risk factors for failure.

## Methods

### Patients

We performed an observational study of all consecutive patients managed with ITTC as first-line treatment for CPPE in the Rennes University Hospital, France, during years 2001–2010. Other aetiologies (i.e. surgery, trauma, mediastinal or sub-diaphragmatic primary infections) were excluded from this study, as well as non-complicated parapneumonic effusions. We included all patients with at least one of the following characteristic for pleural fluid: frank pus (empyema), micro-organisms observed after Gram staining, pH <7.2, glucose level <2.2 mmol/L, loculations or profuse effusion [5], [6].

The study was approved by the Rennes University Hospital Ethics Committee (project approval number 12.52) which waived the informed consent.

### ITTC Protocol

The ITTC protocol was standardized in our department. Thoracentesis were performed at bedside under local anaesthesia (lidocain 1%) using 8- or 10-French disposable pleural needle (Novatech®, La Ciotat, France). Each evacuation was maximal, until no more liquid was aspirated, or until the patient could no longer tolerate the thoracentesis (irrepressible cough, chest pain, or vagal faintness). Thoracentesis were repeated every 1 to 3 days until major decrease of the pleural opacity on chest X-ray and/or until no more pleural fluid could be aspirated. A chest X-ray was performed after each thoracentesis. Intrapleural fibrinolysis protocol was standardized, based on urokinase (Eumedica®, Biarritz, France), 100 000 UI in 50 mL of saline, instilled in the pleural space *via* the pleural needle at the end of thoracentesis. Fibrinolysis was contra-indicated in patients at risk of severe haemorrhagic events, as recommended. The decision of using intrapleural fibrinolysis was left to the physician in charge.

### Failure of Management with ITTC

Failure of ITTC was defined by the need to escalate therapy (i.e. chest tube drainage or thoracic surgery), or death due to sepsis.

### Data Collection

Demographic, clinical, biological and radiological data, especially characteristics of pleural fluid and thoracentesis; and data related to complications and to patients’ vital status were collected from medical records using a standardized questionnaire. Physicians blinded to clinical data reviewed chest X-ray and CT-scan, and collected data on the location and quantity of pleural effusion, the existence of a mediastinal shift, and loculations. Pain was estimated through the use of analogic visual scale when performed, and through the prescription of analgesics.

### Statistical Analysis

Statistical analysis was performed using SAS® version 9.3 software. Two-tailed p-values were reported, with p<0.05 considered as statistically significant.

A descriptive analysis with medians and 25th and 75th percentiles (IQR) for quantitative variables, and absolute and relative frequencies for qualitative variables were performed. The global survival rate of the cohort was estimated using the Kaplan-Meier estimator. The association between the ITTC outcomes and the characteristics of both patients and ITTC procedures was then studied using the Fischer exact test or the Pearson chi-squared test, and the Mann-Whitney U test. Finally, a binomial logistic model was built with the ITTC outcomes as dependent variable, and the overall significant variables associated with the ITTC outcomes in bivariate analysis. Factors with more than 10% of missing data were excluded from the multivariate analysis, and quantitative variables were first categorized in quartile.

## Results

### Patients Characteristics

Seventy-nine consecutive patients were included. Patients and pleural fluids’ characteristics are described in Table 1 and 2. Median age was 54 years (IQR 46–71), and male-to-female sex ratio was 2.59. Most infections were community-acquired (n = 72, 91%). The most frequent comorbidities were alcohol abuse (n = 25, 32%), and neurological disorders (n = 25, 32%). Only 32% of patients did not have any comorbidity.

**Table 1:** Characteristics of patients.

	Total (n = 79)	Success (n = 64)	Failure (n = 15)	p	
**Demographical and Clinical characteristics**					
Median age, years (IQR)	54 (46–71)	56 (45.5–71)	53 (47–72)		0.803
Male gender, n (%)	57 (72%)	45 (70%)	12 (80%)		0.539
Community-acquired infection, n (%)	72 (91%)	59 (92%)	13 (87%)		0.612
Delay symptoms-admission, days (IQR)	10 (5–22)	10 (4.5–21)	8 (5–25)		0.837
Smoker, n (%)	49 (62%)	39 (61%)	10 (67%)		0.774
COPD, n (%)	7 (9%)	6 (9%)	1 (7%)		1.000
Heart disease, n (%)	11 (14%)	9 (14%)	2 (13%)		1.000
Diabetes mellitus, n (%)	8 (10%)	5 (8%)	3 (20%)		0.171
Immunodepression, n (%)	6 (8%)	4 (6%)	2 (13%)		0.319
Alcohol abuse, n (%)	25 (32%)	19 (30%)	6 (40%)		0.540
Cancer, n (%)	13 (16.5%)	10 (16%)	3 (20%)		0.704
Chronic liver disease, n (%)	9 (11%)	5 (8%)	4 (27%)		0.061
Neurological impairment, n (%)	25 (32%)	19 (30%)	6 (40%)		0.540
Non-steroid anti-inflammatory drugs, n (%)	15 (19%)	14 (22%)	1 (7%)		0.279
Corticosteroids >3 weeks, n (%)	9 (11%)	8 (12.5%)	1 (7%)		1.000
Antibiotics initiated before thoracentesis, n (%)	37 (47%)	32 (50%)	5 (33%)		0.268
**Radiological data**					
Right side location, n (%)	46 (58%)	36 (56%)	10 (67%)		0.567
Large effusion (> ½ thorax), n (%)	43 (55%)	33 (52%)	10 (67%)		0.509
Bilateral effusion, n (%)	5 (6%)	5 (8%)	0 (0%)		0.576
Mediastinal shift, n (%)	17 (22%)	10 (16%)	7 (47%)		0.016
Loculations, n (%)	47 (66%)	38 (68%)	9 (60%)		0.557
**Index of diseases severity**					
Respiratory failure, n (%)	12 (15%)	9 (14%)	3 (20%)		0.689
Severe sepsis, n (%)	4 (5%)	3 (5%)	1 (7%)		0.577
Impaired consciousness-Confusion	8 (10%)	6 (9%)	2 (13%)		0.643
Urea, mmol/L (IQR)	5.4 (3.4–8.1)	5.3 (3.4–8.0)	5.9 (3.6–8.2)		0.762
Albumin, g/L (IQR)(†)	22.1 (20.0–24.0)	22.2 (21.7–24.5)	20.9 (19.0–23.0)		0.177

*IQR: Interquartile range; COPD: chronic obstructive pulmonary disease.*

*Quantitative variables are indicated as median (IQR), qualitative variables are indicated as numbers (%). (†) Variables with >10% of missing data.*

**Table 2:** Pleural fluid and microbiology characteristics (first thoracentesis).

	Total (n = 79)	Success (n = 64)	Failure (n = 15)	p	
**Pleural fluid analysis**					
Frank pus (empyema), n (%)	52 (66%)	39 (61%)	13 (87%)		0.073
Protein, g/L (IQR)(†)	46 (41–50)	45 (41–50)	47 (34–48)		0.534
LDH, IU/L (IQR)(†)	4336 (1721–17810)	3932 (1134–14872)	17284 (9452–26068)		0.079
pH (IQR)(†)	7.19 (7.0–7.5)	7.10 (7.0–7.5)	7.50 (7.15–7.75)		0.422
Glucose level, mmol/L (IQR)(†)	1.0 (0.1–3.8)	1.0 (0.1–4.1)	0.6 (0.1–1.1)		0.716
Micro-organisms observed on Gram staining, n (%)	41 (52%)	28 (44%)	13 (87%)		0.003
Positive culture, n (%)	36 (46%)	26 (41%)	10 (67%)		0.088
Positive culture on aerobic atmosphere, n (%)	25 (32%)	20 (31%)	5 (36%)		0.759
Positive culture on anaerobic atmosphere, n (%)	16 (20%)	11 (17%)	5 (36%)		0.036
Polymicrobial culture, n (%)	12 (15%)	8 (12.5%)	4 (27%)		0.227
**Microbiological characteristics**					
Positive blood culture, n (%)	7 (9%)	5 (8%)	2 (14%)		0.612
Positive pneumococcal urine antigen, n (%)	7 (37%)	5 (38.5%)	2 (33%)		1.000
**Identified bacteria**					
Anaerobic bacteria, n (%)	16 (20%)	11 (17%)	5 (33%)		0.171
*Streptococcus milleri*, n (%)	15 (19%)	12 (19%)	3 (20%)		1.000
*Streptococcus pneumoniae*, n (%)	12 (15%)	9 (14%)	3 (20%)		0.689
Other *Streptococcus* sp., n (%)	8 (10%)	5 (8%)	3 (20%)		0.171
*Staphylococcus aureus*, n (%)	3 (4%)	2 (3%)	1 (7%)		0.473
Gram-negative bacteria, n (%)	3 (4%)	2 (3%)	1 (7%)		0.473

*Quantitative variables are indicated as median (IQR), qualitative variables are indicated as numbers (%). (†) Variables with >10% of missing data.*

Loculations were observed on chest X-ray and/or CT scan in 47 (66%) of patients. At least one microbiological documentation was obtained in 45 (57%) patients, from pleural fluid, blood culture, or pneumococcal urinary antigen.

Fifteen patients (19%) were classified as ‘failure’ of the ITTC strategy, including 12 (15%) who were cured by chest tube drainage, and 3 (4%) who finally required surgical drainage.

### Iterative Therapeutic Thoracenteses (ITTC)

ITTC modalities are detailed in Table 3. The median number of thoracenteses was 3 [IQR 2–5]. The median duration of ITTC management was 8 [IQR 4–15] days and the median delay between admission and first therapeutic thoracentesis was 1 [IQR 0–4] day. Blank thoracenteses were observed in 23 (29%) patients.

**Table 3:** Iterative therapeutic thoracentesis (ITTC) modalities and secondary treatments.

	Total (n = 79)		Success (n = 64)	Failure (n = 15)		p
**ITTC modalities**						
Number of thoracentesis (IQR)	3 (2–5)		4 (2–5.5)	3 (2–3)		0.062
Duration of management with ITTC, days (IQR)	8 (4–15)		9 (5–16)	5 (2–7)		0.030
Delay admission –1st thoracentesis, day (IQR)	1 (0–4)		1 (0–4)	1 (0–1)		0.165
Delay symptoms –1st thoracentesis, days (IQR)	12.5 (7–25)		13 (8–27)	9 (6–25)		0.360
Ultrasonography-guided procedure, n (%)	42 (53%)		37 (58%)	5 (33%)		0.149
Urokinase use, n (%)	52 (66%)		42 (66%)	10 (67%)		1.000
Number of urokinase injection (IQR)	2 (1–3)		2 (1–3)	1 (1–2)		0.071
Volume 1^st^ thoracentesis, mL (IQR)	300 (100–450)		200 (100–400)	450 (240–700)		0.009
Volume 2^nd^ thoracentesis, mL (IQR)	200 (140–350)		200 (150–310)	275 (45–400)		0.806
Volume 3^rd^ thoracentesis, mL (IQR)	250 (150–425)		250 (150–375)	425 (100–600)		0.511
Total volume of pleural fluid retrieved, mL (IQR)	875 (500–1600)		847 (500–1545)	1000 (450–1700)		1.000
Blank thoracentesis	23 (29%)		20 (31%)	3 (20%)		0.5332
**Secondary treatments**						
Chest tube drainage, n (% total patients)				12 (15.2%)		
Surgery, n (% total patients)				3 (3.8%)		
**Other treatments**						
Duration of IV antibiotics, days (IQR)	18.5 (11–32)		16.5 (11–29)	31.5 (20–49)		0.012
Total duration of antibiotics, days (IQR)	47 (38–56)	46 (38–53)			49 (44–60)	0.290
Co-amoxiclav use, n (%)	36 (60%)	30 (60%)			6 (67%)	0.720

Ultrasonography-guidance was performed in 42 (53%) patients, and intrapleural fibrinolysis in 52 (66%) patients. Median total volume of pleural fluid removed was 875 mL [IQR 500–1600].

Median duration of antibiotics was 18.5 days [IQR 11–32] for intravenous use, and 47 days [IQR 38–56] when oral treatment was taken into account.

### Complications

Most frequent complications of ITTC included 5 (6%) iatrogenic pneumothoraces and 3 (4%) vagal faintness (Table 4). No pneumothorax required chest tube drainage, and no re-expansion oedema was observed. We did not observe any allergic reaction related to fibrinolytic agent and no hemothorax. However, two major haemorrhagic events occurred (hemoptysis, and gastrointestinal bleeding, one patient each). The median hospital length of stay was 21 days [IQR 14–34]. The median duration of fever was 10 days [IQR 7–15] in total and 7.5 days [IQR 4–13] after the first thoracentesis. Thirteen patients (16%) were admitted in intensive care unit.

**Table 4:** Complications during hospitalisation.

	Total (n = 79)	Success (n = 64)	Failure (n = 15)	p
**Thoracentesis**				
Step 3 analgesic use	22 (29%)	13 (21%)	9 (60%)	0.008
Duration of step 3 analgesic use, days (IQR)	0 (0–1)	0 (0–0)	1 (0–13)	0.019
Vasovagal reaction, n (%)	3 (4%)	3 (5%)	0 (0%)	1.000
Iatrogenic pneumothorax, n (%)	5 (6%)	4 (6%)	1 (7%)	1.000
Confinement to bed, days (IQR)	4 (1–11)	3 (1–6.5)	11.5 (8–21)	0.007
Thromboembolism prophylaxis, days (IQR)	7 (0–17)	5 (0–12)	22 (11–30)	0.005
**General**				
In-hospital death, n (%)	3 (4%)	2 (3%)	1 (7%)	0.473
Hospital stay, days (IQR)	21 (14–34)	21 (13–29)	33 (18–56)	0.036
Fever duration, days (IQR)	10 (7–15)	10 (7–15)	9 (5–32)	0.749
Fever duration after 1^st^ thoracentesis, days (IQR)	7.5 (4–13)	7 (4–13)	9 (4–31)	0.500
ICU admission, n (%)	13 (16%)	5 (8%)	8 (53%)	<10^−3^
Re-hospitalisation rate, n (%)	10 (13.5%)	7 (12%)	3 (21%)	0.388

### Survival

**Figure 1:**
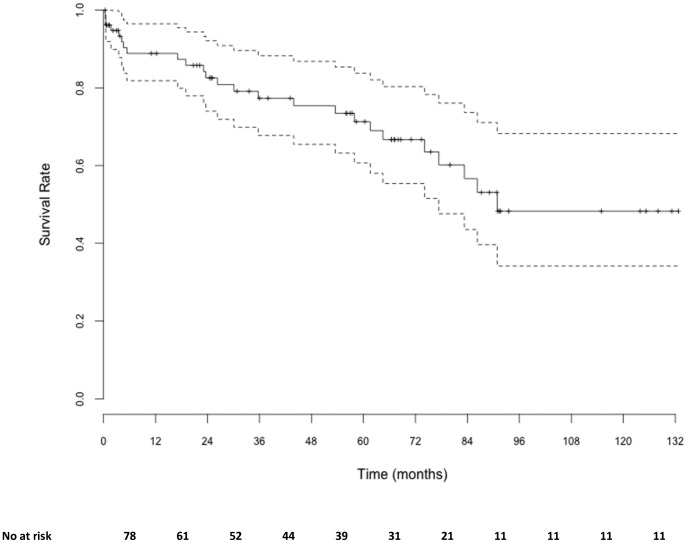
Global survival. Doted lines represent 95% confidence interval.

Three patients (4%) died during hospitalisation and the one year survival rate was estimated at 88.8% [95% CI, 81.8–96.5] (Fig. 1). All early death but one (septic shock) occurred in patients under palliative care due to end-stage comorbidities.

### Predictive Factors of ITTC Failure

Four variables were significantly associated with ITTC failures (Table 5): mediastinal shift (p = 0.016), positive anaerobic culture (p = 0.036), micro-organisms observed after Gram staining in pleural fluid (p = 0.003), and volume of the first thoracentesis ≥450 mL (p = 0.009).

**Table 5:** Factors associated with outcome of iterative therapeutic thoracentesis.

	Univariate	Multivariate	Adjusted odds-ratio	95% CI
Age	NS	NS	–	–
Mediastinal shift	p = 0.016	NS	–	–
Positive culture on anaerobic atmosphere	p = 0.036	NS	–	–
Micro-organisms observed in pleural fluid on Gram staining	p = 0.003	p = 0.017	7.65	1.44–40.67
1^st^ thoracentesis volume ≥450 mL	p = 0.009	p = 0.004	6.97	1.86–26.07

CI: confidence interval; NS: non-significant.

After fitting the data within the logistic model, only two covariates remained associated with the failures of ITTC (Table 5): micro-organisms observed after Gram staining in pleural fluid (OR = 7.65 [95% CI = 1.44–40.67]) and volume of first thoracentesis ≥450 mL [95% CI = 1.86–26.07]).

## Discussion

We analysed 79 consecutive patients with complicated parapneumonic effusion (CPPE) managed with iterative therapeutic thoracentesis (ITTC). Our success rate was 81%, with in-hospital mortality at 4%, and a need for secondary surgery in less than 4% of cases. The median number of thoracentesis needed to achieve cure was 3. Factors associated with failure of ITTC on multivariate analysis were the observation of microorganisms after Gram staining on pleural fluid, and volume of the first thoracentesis ≥450 mL.

Demographic data from our study (median age 54 years, 72% male) and prevalence of comorbidities (68%) are comparable to other studies dealing with CPPE [8]–[13]. Likewise, the high prevalence of empyema (66%), and loculations (66%) are in agreement with previous studies [13], [14]. The relatively low yield of conventional microbiology in pleural fluid is well known [13], [14] and our study highlights the same results, in these settings where patients are frequently on antibiotics before the first thoracentesis (47% of cases in our study). The advent of molecular identification techniques is likely to expand the rate of CPPE with microbiological documentation [15].

In our series, 81% of patients with CPPE could be cured with the combination of antibiotics and ITTC, and did not require chest tube drainage or surgery. The success rates of ITTC are highly variable in the literature, ranging from 2.4% to 100% (Table 6). This variability may be explained by i) differences in patient characteristics; ii) use of heterogeneous protocols (e.g. systematic daily thoracentesis until resolution, versus additional thoracentesis based on clinical and radiological criteria; use of saline irrigation or local antibiotics); iii) the heterogeneity of the criteria used to define success and failure; iv) variability in follow-up duration and methods. The success rate of ITTC in our institution is similar to that of other methods of pleural evacuation in the literature, where success rates ranged from 70% to 94% for chest tube drainage guided by the imaging [16]–[23], and from 71% to 93% for VATS [24]–[26].

**Table 6:** Success rates of iterative therapeutic thoracentesis in clinical studies: a literature review.

Author	Characteristics	Number ofpatients	Type of pleuraleffusion	Success n (%)	Mortality n (%)
Viana *et al.* [39]	Monocentric 1964–1968	41	NR	1 (2.4%)	8 (19.5%)
Benfield *et al.* [40]	Monocentric 1968–1978	24	NR	8 (33.3%)	3 (12.5%)
Lemmer *et al.* [41]	Monocentric 1978–1982	4	NR	3 (75%)	1 (25%)
Mandal *et al.* [42]	Monocentric 1972–1984	28	50% CPPE	28 (100%)	0 (0%)
Wehr *et al.* [28]	Monocentric 1974–1984	27	NR	6 (22.2%)	2 (7.4%)
Storm *et al.* [43]	Monocentric 1984–1989	51	100% CPPE and empyema	48 (94.1%)	4 (7.8%)
Ferguson *et al.* [8]	Multicentric 1986–1990	46	100% empyema	19 (41%)	3 (6.5%)
Simmers *et al.* [44]	Monocentric 1999	29	100% CPPE and empyema	25 (86%)	4 (14%)
Letheulle *et al.*	Monocentric 2013	79	100% CPPE and empyema	64 (81%)	4 (12%)

NR: not reported; CPPE: complicated parapneumonic effusions.

Our criteria for failure were death, and/or therapeutic escalation, from ITTC to more invasive procedures (i.e. chest tube drainage or thoracic surgery). The most recent studies [13], [14] defined success as the resolution of CPPE without surgery. With this criterion, our rate of success would have been 96% in our series. In comparison with previous studies relating ITTC, the need for surgery in our study was lower than in Storm *et al.* [27] and Wehr *et al.* [28] series (respectively, 6% and 18%). In comparison with the MIST studies, the need for surgery in our study is lower than in MIST1 [13] (16% in the intervention group) and similar to MIST2 [14] (4%). Overall these data suggest that surgery can be avoided in most cases of CPPE [29], which may be preferable given the high prevalence of comorbidities in these populations [8], [30], [31].

In our series, the in-hospital mortality was 4%, with a mortality of 12% at one year. Those results are in accordance with epidemiologic data [30], [32].

In our study, two parameters were independently associated with failure: microorganisms observed on Gram stain of pleural fluid, and first thoracentesis volume ≥450 mL. These two items are available at the beginning of the management and could therefore be used as a guide for closer monitoring, or earlier switch to more invasive strategy. Of note, the observation of micro-organisms after Gram stain on pleural fluid was not affected by antibiotic use prior to first thoracentesis (p = 0.320), and first thoracentesis volume was not correlated to imaging findings such as the estimated size of effusion (p = 0.066), the presence of mediastinal shift (p = 0.203), loculations (p = 1.000), and was not affected by ultrasound guidance (p = 0.790). Finally, as observed in the MIST studies [13], [14], fibrinolytic use was not associated with improved outcome. To our knowledge, only one study analysed factors associated with ITTC failure [8], and found that an estimated pleural volume ≥40% of the thorax was associated with worse outcome. The factors identified as significantly predictive of chest tube drainage failure to date are the presence of empyema, and loculations [12], [30].

In our series as in others, ITTC was well tolerated. Pain during thoracentesis has been reported in 15% to 28% of cases [33]–[37]. WHO step 3 analgesics were used in 29% of patients in our series, for less than 24 hours (IQR = 1). Vasovagal reactions have been reported in 2% to 4% of thoracentesis [36], [38], as in our series (4%). Pneumothorax complicated 4 to 30% of thoracentesis in the literature, and required chest tube drainage in 20% to 50% of cases [33]–[36]. The rate of post-ITTC pneumothorax was 6% in our series, and never required chest tube drainage. In addition, no hemothorax was reported despite the use of intrapleural fibrinolytics in two thirds of cases. These low rates of local complications may be related to the systematic use of atraumatic needles, and the large experience accumulated over years, through the protocolized management of ITTC.

The main limitations of our study are inherent to its design (retrospective, observational, and monocentric). Failure was defined as escalation, which was not protocolized, and left to the physician in charge, based on the combination of clinical, microbiological, and imaging data. Hence, classification biases may have occurred (e.g. patients classified as ‘failure’ could have been successfully controlled with ITTC). This means that our estimated 81% success rate is conservative. The rate of missing data did not exceed 10% except for a few variables which were not included in multivariate analysis.

The main caveats of our strategy based on ITTC are the following: i) its reliance on experimented staff, which means that these encouraging results may not be replicated in every institutions; ii) the significant duration of hospital stay (median, 21 days in our study), although we are currently reducing hospital stay by earlier discharge, and the development of ambulatory care units; iii) the need to repeat the procedure (median, three times in our series), which means that ITTC may be more time-consuming for physicians, globally, than chest tube drainage.

Our study has several strengths. To our knowledge, this study included the largest number of patients treated with ITTC (Table 6).

Our selection criteria enabled the constitution of an homogeneous group of patients with CPPE requiring pleural drainage according to current recommendations [5], [6]. Finally, throughout the study period, standardized protocols for thoracentesis and pleural fibrinolysis were applied.

In conclusion, our study demonstrates that ITTC is safe and effective as first-line treatment of CPPE. However, the design of our study (observational, monocentric), precludes comparison of ITTC with other common therapeutic strategies, such as chest tube drainage or surgery. The identification of variables available with the first thoracentesis and independently associated with failure of the ITTC strategy led us to implement the following protocol: The first thoracentesis is performed for both diagnostic and therapeutic purposes. If micro-organisms are observed after Gram stain on pleural fluid and/or if the volume evacuated during this first procedure is ≥450 mL, patients will be closely monitored, and chest tube drainage or VATS will be considered in case of uncontrolled sepsis at day 4. In the absence of both failure criteria (microorganisms observed on Gram stain of pleural fluid, and first thoracentesis volume ≥450 mL), ITTC was associated with a success rate of 97% in our series. A randomized study comparing ITTC *vs.* chest tube drainage would be required to establish the optimal first-line treatment of CPPE.
